# The RU_SATED as a measure of sleep health: cross-cultural adaptation and validation in Chinese healthcare students

**DOI:** 10.1186/s40359-023-01203-5

**Published:** 2023-07-05

**Authors:** Runtang Meng, Lu Dong, Joseph M. Dzierzewski, Stefanos Mastrotheodoros, Menglu Cao, Bilin Yu, Jue Wang, Boxiong Gong, Jingjing Li, Karen Spruyt

**Affiliations:** 1 School of Public Health, Hangzhou Normal University, Hangzhou, 311121 Zhejiang China; 2 Engineering Research Center of Mobile Health Management System, Ministry of Education, Hangzhou, Zhejiang China; 3 Department of Behavioral and Policy Sciences, RAND Corporation, Santa Monica, CA USA; 4 Department of Psychology, Virginia Commonwealth University, Richmond, VA USA; 5 The National Sleep Foundation, Washington, DC USA; 6 Department of Psychology, University of Crete, Rethymno, Greece; 7 Department of Youth and Family, Utrecht University, Utrecht, The Netherlands; 8 Faculty of Psychology, Southwest University, Chongqing, China; 9 Student’s Mental Health Center, Sichuan Technology and Business University, Chengdu, Sichuan China; 10 School of International Journalism and Communication, Beijing Foreign Studies University, Beijing, China; 11 School of Basic Medical, Shanghai University of Traditional Chinese Medicine, Shanghai, China; 12 Department of Oncology, Taihe Hospital, Hubei University of Medicine, Shiyan, Hubei China; 13 Department of Behavioral Sciences and Health Education, Rollins School of Public Health, Emory University, Atlanta, GA USA; 14 Université de Paris, NeuroDiderot, INSERM, Paris, France

**Keywords:** RU_SATED, Sleep health, Cross-cultural adaptation, Validation, Psychometrics, Longitudinal

## Abstract

**Background:**

The RU_SATED scale is a multidimensional instrument measuring sleep health, consisting of Regularity, Satisfaction, Alertness, Timing, Efficiency, Duration dimensions. We adapted and validated the Chinese RU_SATED (RU_SATED-C) scale.

**Methods:**

The RU_SATED-C scale was developed through a formal linguistic validation process and was validated in an observational longitudinal survey design. Healthcare students completed the RU_SATED scale, Sleep Quality Questionnaire, and Patient Health Questionnaire-4 among two sites of Hangzhou and Ningbo, China. Psychometric assessments included structural validity, longitudinal measurement invariance, convergent and divergent validity, internal consistency, and test–retest reliability.

**Results:**

A total of 911 healthcare students completed the RU_SATED-C scale at baseline (Time 1, T1) and follow-up (Time 2, T2) with an average time interval of 7 days + 5.37 h. Confirmatory factor analysis (CFA) confirmed a single-factor model and resulted in an acceptable model fit. The two-factor model previously found in the Japanese version fit better than the one-factor model, whereas the one-factor model fit had a better fit than the two-factor model found in the English version. Longitudinal CFA resulted in negligible changes in fit indices for four forms of increasingly restrictive models and supported that a single-factor model was equivalent over time. The data also endorsed longitudinal measurement invariance among the two-factor models found in the English and Japanese samples. The RU_SATED-C scale total score displayed a moderately strong negative correlation with sleep quality; however, negligible associations were observed with anxiety and depression. Ordinal Cronbach’s alpha and Ordinal McDonald's omega at T1 and T2 ranged from suboptimal to acceptable. The RU_SATED-C scale and all items were significantly correlated across time intervals.

**Conclusion:**

The RU_SATED-C scale is an easy-to-use instrument with potentially valid data for the measurement of multidimensional sleep health. Use of the RU_SATED-C scale can help raise awareness of sleep health and could pave the way for important efforts to promote healthy sleep.

**Supplementary Information:**

The online version contains supplementary material available at 10.1186/s40359-023-01203-5.

## Introduction

Better sleep is a cornerstone of better health. To date, sleep health is recognized as a major global public health concern; thus, improving sleep health is a necessary step toward achieving better health [1]. While significant resources have been invested in individual and population-level interventions to remedy unhealthy lifestyle factors such as nutrition, exercise, and smoking control, programs concentrating on sleep health have been notably scarce [2]. Sleep health has been defined as “a multidimensional pattern of sleep-wakefulness, adapted to individual, social, and environmental demands, that promotes physical and mental well-being” [3]. The sleep health framework was developed based on an extensive review of the scientific literature, including a review of specific dimensions of sleep and their association with numerous health outcomes, providing a comprehensive framework for examining sleep health. This multidimensional sleep health framework differs from traditional operationalizations of sleep in medicine in that it does not focus on identifying and treating sleep disorders; instead, sleep health promotes a positive framework that views sleep as a multidimensional construct considering positive attributes of sleep along important dimensions—sleep duration, sleep continuity or efficiency, timing, alertness/sleepiness, satisfaction/quality, and regularity—that are associated with physical and mental health. Alternatively, sleep health is broadly defined as a pattern of sleep that is associated with optimal physical and mental health, and is not merely the absence of a sleep disorder, encompassing sleep duration and quality in non-disordered sleepers [4].

Assessing and promoting multidimensional sleep health may offer potential benefits [2, 3, 5–9]. First, consistent with the World Health Organization’s (WHO) definition of health, the concept of sleep health broadens key dimensions of good sleep and enables individuals to comprehensively quantify and modify the level of sleep health. Second, the conceptual framework of sleep health provides useful building blocks and frameworks that facilitate developing new sleep health instruments, as a foundation for adding additional domains. Third, the sleep health framework avoids simply dichotomizing the sleep conditions of individuals into healthy and unhealthy by capturing graduations in sleep, noting that the sleep health of individuals exists on a continuum. Finally, identifying and measuring sleep health instead of only assessing and treating sleep disorders may increase awareness, optimize personalized sleep recommendations, and enhance evidence-based self-management of sleep behaviors, more significantly, allowing for earlier interventions to prevent the adverse downstream effects of suboptimal sleep.

Sleep can be assessed via objective and subjective measures including self-report questionnaires or sleep diaries, actigraphy, and home or laboratory polysomnography [3]. While there have been numerous instruments that assess sleep disturbance or sleep quality in clinical and research settings, instruments that measure sleep health are rare. A short, practical self-report instrument for the measurement of sleep health called the SATED (Satisfaction, Alertness, Timing, Efficiency, Duration) (v1.0) scale was proposed in 2014 [3], with subsequent expansion to include an additional dimension/item assessing Regularity. The current instrument is now called the RU_SATED (v2.0) scale [3]. Another sleep health assessment instrument was developed by the National Sleep Foundation—Sleep Health Index (SHI) [10]. The SHI is a 12-item instrument designed to capture three discrete dimensions of sleep health: duration, quality, and disorders. Noteworthy, the RU_SATED scale has a richer conception of sleep health, and is half the length of the SHI, while including more theoretical dimensions of sleep health. These six dimensions of the RU_SATED scale are appropriate indicators of sleep health for several reasons. First, each has been associated with important health outcomes, albeit with somewhat different outcomes for each dimension. Second, they can each be expressed in positive terms, i.e., we can characterize their “better” directions. This is not to say that these dimensions are all unidirectional. It is also important to acknowledge that, while these dimensions can be expressed in positive terms, the supporting studies largely focus on their negative directions and consequences; there have been few studies specifically examining the potential benefits of good sleep.

Poor sleep is common among healthcare students, with prevalence estimates suggesting higher rates of poor sleep than in non-healthcare students and the general population [11–14]. A 2022 meta-analysis reported that the prevalence of sleep problems among healthcare students was close to thirty percent in China [15]. The domains of sleep health that are typically poor in healthcare students due to academic overload and rigorous training are: insufficient sleep duration, poor sleep quality, and excessive daytime sleepiness amongst others [11, 13, 14]. The dire situation for healthcare students requires urgent attention and effective intervention, such as regular monitoring and screening of poor sleep health. Healthcare students, therefore, comprise an important and interesting population in which to test the RU_SATED scale.

To date, the RU_SATED scale has been cross-culturally adapted and validated in at least five languages: Portuguese (2018) [16], English (2019) [9], Spanish (2020) [17], French (2021) [18], and Japanese (2022) [19]. In the current study, we developed the Chinese version of the RU_SATED (RU_SATED-C) scale and conducted a longitudinal observational design in a sample of healthcare students in China. The primary study aims were to (i) develop a Chinese version of the RU_SATED scale and (ii) assess the main measurement properties of the RU_SATED-C scale: structural validity, longitudinal measurement invariance, convergent and divergent validity, internal consistency, and test–retest reliability.

## Methods

### Linguistic validation of the Chinese RU_SATED (RU_SATED-C) scale

Using the formal procedure for linguistic validation, the original RU_SATED (v2.0) scale was translated into Chinese following Mapi instructions [20], including translation by two separate translators, qualitative interviews to determine people's understanding of the questions in the new language (i.e., Chinese), and back-translation by two other translators. The linguistic validation process is essential to ensure that the RU_SATED (v2.0) scale is actually measuring what it was intended to measure in the newly translated language.

*Step 1* Preparation: Initial planning and actions carried out before the translation process began included conceptual analysis of the original questionnaire and application for approval to use the original questionnaire. After obtaining permission from the original author (Prof. Daniel J. Buysse, DJB) of the RU_SATED scale, an e-contract was signed with the University of Pittsburgh for the preparation of the Chinese version of the RU_SATED scale.

*Step 2* Forward translation: The original RU_SATED scale was translated into Chinese independently by two Chinese native speakers, a psychologist (co-author, MC), and a linguist (BY) with a high level of fluency in both English and Chinese. A panel of five local clinical and research experts (MC, BY, JW, BG, and RM) checked and compared the two translations to create the preliminary initial translated form of the scale.

*Step 3* Backward translation: The back-translation into English was undertaken by two independent highly proficient bilingual English-Chinese speakers (i.e., a behavioral scientist and clinical psychologist [LD] and a behavioral scientist and physician [JL]), and was made independently of the forward translation. The original author (DJB) reviewed the two back-translations, which were rated as satisfactory.

*Step 4* Pilot Testing: Eight Chinese healthcare students were surveyed to see whether they could understand the meaning of the translated items, instrument instructions, and answer choices. Pilot testing revealed that no explanations were required, with all eight individuals confirming full understanding of the RU_SATED-C scale.

*Step 5* Proofreading and finalization: The research team (RM, LD, JL, MC, BY, JW, BG, and DJB) involved in the forward translation, consolidation, and backward translation processes evaluated the pre-final version of the scale and confirmed the equivalence between the Chinese and English versions. The final Chinese RU_SATED scale was delivered to the original author (DJB) and is housed electronically at the University of Pittsburgh.

### Participants and procedures

For this validation study, routinely collected data were available from two sample sites (Hangzhou and Ningbo, China) and contained an assessment of sleep using the below three measures from December 2020 until January 2021. The trained investigators were responsible for the conduct of the survey and its onsite quality control. Self-administered paper-and-pencil survey was centralized at recess or evening self-study. Healthcare students were recruited by applying a stratified random sampling approach based on their academic years and majors [21]. Inclusion criteria: individuals who were able to read simplified Chinese and communicate in Mandarin. Exclusion criteria: 1) people who were reluctant to participate; 2) those who had difficulty understanding the study procedures. Given that a retest interval of two to 14 days is usually adequate [22, 23] and reproducibility of health status measures intended for longitudinal use may best be measured at intervals of 1–2 weeks [24]. 976 healthcare students responded to the baseline assessment (Time 1, T1) and 951 completed a follow-up assessment approximately 7 days later (Time 2, T2). A total of 911 questionnaires were matched by student ID at two time points. Each participant received 2 CNY (around 0.30 US dollars) upon completion of the study as compensation for their time.

### Measures

#### RU_SATED scale

Sleep health was assessed using the RU_SATED (Regularity, Satisfaction, Alertness, Timing, Efficiency, Duration) scale, consisting of six key dimensions of sleep health that are consistently associated with various health outcomes [3]. The scale consists of six items/dimensions of sleep health and queries about sleep during the previous month. Each item is scored from 0 to 2 on a three-point Likert scale, with 0 for “never” or “rarely,” 1 for “sometimes,” and 2 for “usually” or “always.” Scoring entails summing the scores of the individual items, with total scores ranging from 0 (poor sleep health) to 12 (good sleep health).

#### Sleep quality questionnaire (SQQ)

Sleep quality was measured by the Sleep Quality Questionnaire (SQQ) [25]. This questionnaire evaluates two components—daytime sleepiness (four items) and sleep difficulty (six items)—of sleep quality in the last month. Each item is scored from 0 (strongly disagree) to 4 (strongly agree) on a five-point Likert scale. The overall SQQ score ranges from 0 to 40, with higher scores indicating poorer sleep quality. Psychometric data for the Chinese version of the Sleep Quality Questionnaire (SQQ-C) reveal adequate measurement properties in multi-site studies [21, 26–28].

#### Patient health questionnaire-4 (PHQ-4)

A self-report version of the Primary Care Evaluation of Mental Disorders (PRIME-MD) called the Patient Health Questionnaire (PHQ) was developed and validated in two large studies for use with general adult samples [29]. The PHQ-4 is a validated measure of mental health symptoms consisting of the first two items of the PHQ-9 and the GAD-7, respectively [30]. Each item is scored from 0 (not at all) to 3 (nearly every day). The total score ranges from 0 to 6, with a higher score indicating greater severity of anxiety or depression over the last two weeks. The Chinese version of the PHQ-4 (PHQ-4-C) and its instruction manual are publicly available and no permission is required for use [31].

### Statistical analysis

#### Data preparation

Data were checked for data entry errors, missing data, or the presence of extreme outliers. Frequencies (%) were calculated for categorical variables, whereas means and standard deviations were computed for continuous variables. Multivariate normality was assessed via skewness and kurtosis. Data analyses were performed with JASP (v.0.16.1) and R (v.4.1.2). The packages “*naniar* v 1.0.0” [32], “*MVN* v.5.9” [33], “*lavaan* v.0.6-9” [34], “*semTools* v.0.5-5” [35], “*irr* v.0.84.1” [36], and “*ufs* v.0.5.2” [37] under RStudio were utilized to conduct the missing value analysis, multivariate normality tests, confirmatory factor analysis (CFA), longitudinal CFA (LCFA), intraclass correlation coefficient (ICC), and Cronbach’s alpha as well as McDonald’s omega. After missing value analysis, of the 911 participants, 898 (98.6%) had no missing data, while 13 (1.43%) had some missing data. Of the total 12 RU_SATED-C scale items (T1 and T2) missingness ranged from 0.11% to 0.44%. Missingness was therefore considered negligible, and listwise deletion was applied for factor analysis (i.e., structural validity and longitudinal measurement invariance) and reliability analysis (i.e., internal consistency and test–retest reliability). In other analyses (N = 911), convergent and divergent validity and reliability for other measures, missing data was replaced by the mean or median of observed values given that missing data rates did not exceed 10% [38, 39] or 5% [40]. We assessed the below measurement properties of the measures, adhering to the COnsensus-based Standards for the selection of health Measurement INstruments (COSMIN) taxonomy and guideline [41, 42].

#### Structural validity

Structural validity measures the degree to which the scores of an instrument are an adequate reflection of the dimensionality of the construct measured [42]. The structural validity of the RU_SATED-C scale was assessed by CFAs. Because the six items are supposed to measure one construct (sleep health), we expected that all items would load on a single factor [3], similar to that of findings in the Portuguese, Spanish, and French samples [16–18]. The single-factor structure of the RU_SATED-C scale was evaluated across two points in time independently (T1 and T2; a cross-sectional CFA at each time point), as well as through a LCFA approach. We applied the mean and variance adjusted diagonally weighted least squares (DWLS) estimator based on the polychoric correlation matrix to examine unidimensionality, given those responses to items in the RU_SATED-C scale are ordinal [43, 44]. In addition to the one-factor model, we examined the fit of the two-factor models that were found in the English and Japanese samples [9, 19].

Model fit indices include the chi-squared test statistic and its associated degrees-of-freedom (*df*) and *p*-value [40]. However, considering that the chi-squared test is known to be very sensitive to large sample sizes, we also included additional relevant fit indices: comparative fit index (CFI), Tucker–Lewis index (TLI), root means square error of approximation (RMSEA) and its corresponding 90% confidence interval. Scaled fit indices instead of unscaled indices were reported in this paper because the former is considered more precise [45]. Following the recommended guidelines, we considered acceptable model fit if CFI ≥ 0.90, TLI ≥ 0.90, and RMSEA ≤ 0.08 [40, 46]; good model fit if CFI ≥ 0.95 or TLI ≥ 0.95, and RMSEA ≤ 0.06 [41, 47].

#### Longitudinal measurement invariance

Following confirmation of the single-factor and two-factor structure of the RU_SATED-C scale, we explored longitudinal measurement invariance (LMI) in the matched sample (N = 898) across time. LCFA was used to examine four forms of increasingly restrictive invariance: configural invariance (same pattern of free loadings), metric or weak invariance (common loadings over time), scalar or strong invariance (common loadings and intercepts over time), and strict or residual invariance (common loadings, intercepts, and residual variances over time). The fit of two nested models can be compared by taking the difference of the fit indices. However, the scaled chi-square difference suffers from the same issues of significance with large sample sizes as the minimum fit function statistic [48]. Hence, we focused on changes in model fit according to CFI, TLI, and RMSEA when the scaled chi-square difference was significant [48]. Following the recommended cut-off criteria, we considered an acceptable model fit for more restrictive invariant models in the following circumstances: ΔCFI ≤ 0.010, ΔTLI ≤ 0.010, and ΔRMSEA ≤ 0.015 [49]. If at least two out of three changes in fit indices meet the cut-off criteria, we considered that longitudinal measurement invariance held [50].

#### Convergent and divergent validity

For assessing convergent and divergent validity, we hypothesized that the RU_SATED-C scale total score would have a moderately strong negative correlation (− 0.50 < **r** < − 0.30, Spearman) with the SQQ-C, given that both instruments measure sleep-related constructs, and a weak negative correlation (− 0.30 < *r* <  0, Spearman) with the PHQ-4-C, due to the theoretically distinct nature of sleep and mental health constructs [41].

#### Internal consistency

Internal consistency measures the degree of interrelatedness among measure items [42]. Internal consistency of the RU_SATED-C scale was determined by calculating ordinal Cronbach’s alpha and McDonald's omega to accommodate categorical data [51]. Values greater or equal to 0.70 was considered sufficient evidence for internal consistency [52].

#### Test–retest reliability

Test–retest reliability reflects the consistency in measurement taken by the same instrument, on the same subjects, under the same or very similar conditions [53]. ICC estimated by a two-way mixed model was used to evaluate test–retest reliability of the RU_SATED-C scale. An ICC < 0.40 was considered poor, 0.40 ≤ ICC < 0.60 fair, 0.60 ≤ ICC < 0.75 good, and ICC ≥ 0.75 excellent [54].

## Results

### Descriptive

We analyzed final data from the matched sample of 911 healthcare students (complete data response rate 93.34% and 95.79% for T1 and T2, respectively). The average time interval between T1 and T2 was 7 days + 5.37 h. The mean age of participants was 19.66 ± 1.45 years, ranging from 17 to 31 years. Additional descriptive information is presented in Table 1. Means, standard deviations, skewness, kurtosis, and amount of missing data at T1 and T2 are presented in Additional file 1: Table S1.

**Table 1:** Descriptive characteristics of study cohort (N = 911)

Characteristics	n (%)
*Gender*	
Female	736 (80.790)
Male	175 (19.210)
*Age*	
≤ 18	200 (21.954)
19	217 (23.820)
20	315 (34.577)
21	118 (12.953)
≥ 22	61 (6.696)
*Major*	
Clinical medicine	121 (13.282)
Preventive medicine	96 (10.538)
Nursing	270 (29.638)
Pharmacy	94 (10.318)
Health policy and management	139 (15.258)
Health services and management	81 (8.891)
Midwifery	69 (7.574)
Stomatology	41 (4.501)
*Academic stage*	
Junior college students	316 (34.687)
Undergraduate	554 (60.812)
Postgraduate	41 (4.501)

### Structural validity and longitudinal measurement invariance

The CFA at each time point resulted in an acceptable fit for the single-factor model [CFI = 0.958, TLI = 0.929, RMSEA = 0.054 (0.035, 0.075) and CFI = 0.967, TLI = 0.945, RMSEA = 0.058 (0.039, 0.079)] at T1 and T2, respectively (Table 2). The fit indices were CFI = 0.976, TLI = 0.956, RMSEA = 0.043 (0.021, 0.066) and CFI = 0.974, TLI = 0.952, RMSEA = 0.055 (0.035, 0.077)] at T1 and T2, respectively, indicating good fit of the two-factor model found in the Japanese samples. Similarly, the fit indices were CFI = 0.957, TLI = 0.919, RMSEA = 0.058 (0.038, 0.080) and CFI = 0.966, TLI = 0.937, RMSEA = 0.063 (0.043, 0.084)] at T1 and T2, respectively, indicating acceptable fit of the two-factor model found in the English samples. After the scaled chi-squared difference test, we compared the one-factor and two-factor models fit at the same time point. The two-factor model in the Japanese version fit outperformed that of the one-factor model and the models differed substantially, and the one-factor model fit was superior to that of the two-factor model in the English version and yet the model difference was negligible.

**Table 2:** Fit indices for alternative models of the RU_SATED-C at T1 and T2 (N = 898)

Model	*χ*^*2*^ (*df*)	*P*	CFI	TLI	RMSEA (90% CI)	Scaled Chi-squared difference test	
						Δ*χ*^*2*^ (Δ*df*)	*P*
One-factor (T1)	32.974 (9)	< 0.001	0.958	0.929	0.054 (0.035, 0.075)		
One-factor (T2)	36.421 (9)	< 0.001	0.967	0.945	0.058 (0.039, 0.079)		
Two-factor (T1, Japanese)	21.317 (8)	< 0.001	0.976	0.956	0.043 (0.021, 0.066)	13.399 (1)	< 0.001
Two-factor (T2, Japanese)	29.623 (8)	< 0.001	0.974	0.952	0.055 (0.035, 0.077)	7.579 (1)	0.006
Two-factor (T1, English)	32.407 (8)	< 0.001	0.957	0.919	0.058 (0.038, 0.080)	0.656 (1)	0.418
Two-factor (T2, English)	36.313 (8)	< 0.001	0.966	0.937	0.063 (0.043, 0.084)	0.155 (1)	0.694
Threshold	N/A	> 0.05	> 0.900	> 0.900	< 0.080		

Report “Used/Standard” fit indices due to use of listwise deletion; *df*, degrees of freedom; CFI, comparative fit index; TLI, Tucker–Lewis index; RMSEA, root mean square error of approximation; CI, confidence interval; T1, Time 1; T2, Time 2; N/A, not applicable

The LCFA provided strong evidence for invariance (Table 3). Configural invariance was supported by fit indices meeting requirements for excellent model fit [CFI = 0.987, TLI = 0.982, RMSEA = 0.047 (0.038, 0.056)] for the single-factor model, [CFI = 0.991, TLI = 0.986, RMSEA = 0.041 (0.032, 0.051)] for the two-factor model in the Japanese version, [CFI = 0.987, TLI = 0.980, RMSEA = 0.051 (0.041, 0.060)] for the two-factor model in the English version, respectively. Successively stricter constraints on factor loadings (metric), loadings and intercepts (scalar), and loadings, intercepts, and residual variances (strict) revealed that all three invariance models (metric, scalar, and strict measurement invariance) were supported with negligible changes in fit indices across time (all ∆CFI ≤ 0.010, ΔTLI ≤ 0.010, and ∆RMSEA ≤ 0.015). Hence, all four forms of longitudinal measurement invariance among the single-factor model and the two-factor models in Japanese and English versions were supported.

**Table 3:** Longitudinal measurement invariance of the RU_SATED-C across time (N = 898)

Hypothesis	*χ*^*2*^ (*df*)	*P*	Scaled Chi-squared difference test		CFI	ΔCFI	TLI	ΔTLI	RMSEA (90% CI)	ΔRMSEA
			Δ*χ*^*2*^ (Δ*df*)	*P*						
*One-factor*										
Configural	139.541 (47)	< 0.001			0.987		0.982		0.047 (0.038, 0.056)	
Metric	143.861 (52)	< 0.001	4.899 (5)	0.428	0.988	0.001	0.984	0.002	0.044 (0.044, 0.053)	− 0.003
Scalar	165.746 (57)	< 0.001	31.812 (5)	< 0.001	0.985	− 0.003	0.983	− 0.001	0.046 (0.038, 0.054)	0.002
Strict	185.003 (63)	< 0.001	25.691 (6)	< 0.001	0.983	− 0.002	0.983	0.000	0.046 (0.039, 0.054)	0.000
*Two-factor (Japanese)*										
Configural	106.216 (42)	< 0.001			0.991		0.986		0.041 (0.032, 0.051)	
Metric	110.776 (46)	< 0.001	5.416 (4)	0.247	0.991	0.000	0.987	0.001	0.040 (0.030, 0.049)	− 0.001
Scalar	123.231 (50)	< 0.001	18.179 (4)	0.001	0.990	− 0.001	0.987	0.000	0.040 (0.031, 0.049)	0.000
Strict	146.819 (56)	< 0.001	33.438 (6)	< 0.001	0.988	− 0.002	0.985	− 0.002	0.043 (0.034, 0.051)	0.003
*Two-factor (English)*										
Configural	138.104 (42)	< 0.001			0.987		0.980		0.051 (0.041, 0.060)	
Metric	138.546 (46)	< 0.001	0.535 (4)	0.970	0.987	0.000	0.982	0.002	0.047 (0.038, 0.057)	− 0.004
Scalar	154.578 (50)	< 0.001	23.596 (4)	< 0.001	0.986	− 0.001	0.981	− 0.001	0.048 (0.040, 0.057)	0.001
Strict	164.531 (56)	< 0.001	14.025 (6)	0.029	0.985	− 0.001	0.983	0.002	0.046 (0.038, 0.055)	− 0.002
Threshold	N/A	> 0.05	N/A	> 0.05	> 0.90	< 0.010	> 0.90	< 0.010	< 0.08	< 0.015

Report “Used/Standard” fit indices due to use of listwise deletion; *df*, degrees of freedom; CFI, comparative fit index; TLI, Tucker–Lewis index; RMSEA, root mean square error of approximation; CI, confidence interval; N/A, not applicable

### Convergent and divergent validity

**Fig. 1:**
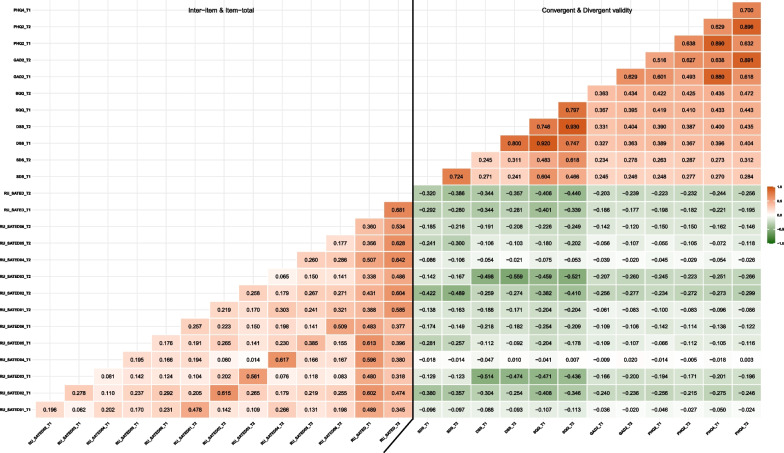
Inter–item and item–total, convergent and divergent correlations between the RU_SATED-C scale, SQQ-C and PHQ-4-C (N = 911). *Note*: Spearman correlations; T1, Time 1; T2, Time 2; RU_SATED, Regularity, Satisfaction, Alertness, Timing, Efficiency, Duration; SQQ, Sleep Quality Questionnaire; DSS, Daytime Sleepiness Subscale; SDS, Sleep Difficulty Subscale; PHQ-4, Patient Health Questionnaire-4; GAD-2, Generalized Anxiety Disorder-2; PHQ-2, Patient Health Questionnaire-2

The total scores for the RU_SATED-C scale at T1 and T2 were 8.286 ± 2.148 and 8.375 ± 2.230, respectively. The total scores for the SQQ-C at T1 and T2 were 18.058 ± 6.265 and 17.903 ± 6.343, respectively. The total scores for the PHQ-4-C at T1 and T2 were 3.501 ± 2.214 and 3.318 ± 2.016, respectively. The Spearman correlations of the total SQQ-C scores with the total RU_SATED-C scale scores were − 0.401 and − 0.440 at T1 and T2, respectively (all *P* < 0.001), providing support for convergent validity. The Spearman correlations of the total PHQ-4-C scores with the total RU_SATED-C scale scores were − 0.221 and − 0.256 at T1 and T2, respectively, (all *P* < 0.001), providing support for divergent validity. The correlation matrix of the RU_SATED-C scores on inter-item and item-total, and with the SQQ-C and the PHQ-4-C scores on subscales and global scale, are presented in Additional file 1: Table S2. Spearman correlations to establish convergent and divergent validity are shown in Fig. 1.

### Internal consistency and test–retest reliability

Ordinal Cronbach’s alpha at T1 and T2 were 0.670 and 0.722, respectively. Ordinal McDonald's omega at T1 and T2 were 0.676 and 0.725, respectively. Both metrics are suggestive of suboptimal levels of internal consistency (Table 4). ICC analyses showed that the RU_SATED-C scale and items were significantly correlated across time intervals (ICC = 0.354–0.683), suggestive of fair to good test–retest reliability, with the exception of item 5 which demonstrated poor test–retest reliability (Efficiency) (Table 4).

**Table 4:** Internal consistency and test–retest reliability for the RU_SATED-C at T1 and T2 (N = 898)

	Total/items	SEM	
		T1	T2
Ordinal Cronbach's Alpha			
T1	0.670 (0.637, 0.704)		
T2	0.722 (0.693, 0.750)		
Ordinal McDonald's Omega			
T1	0.676 (0.643, 0.708)		
T2	0.725 (0.698, 0.753)		
ICC (T1, T2)	0.683 (0.646, 0.716)	1.216	1.259
01. Do you go to bed and get out of bed at about the same time (within one hour) every day?	0.464 (0.411, 0.514)	0.403	0.406
02. Are you satisfied with your sleep?	0.621 (0.579, 0.660)	0.406	0.398
03. Do you stay awake all day without dozing?	0.561 (0.513, 0.604)	0.404	0.411
04. Is the middle of your sleep between 2:00 a.m. and 4:00 a.m.?	0.616 (0.574, 0.655)	0.506	0.476
05. Do you spend less than 30 min awake at night? This includes the time it takes to fall asleep plus awakenings during sleep.	0.354 (0.296, 0.410)	0.597	0.593
06. Do you sleep between 6 and 8 h per day?	0.531 (0.482, 0.576)	0.318	0.331

Point estimate and 95% confidence interval; Estimates on Cronbach's Alpha and McDonald's Omega assuming ordinal level; T1, Time 1; T2, Time 2; ICC, intraclass correlation efficient; SEM, standard error of measurement; SEM = SD × sqrt (1-ICC); SD, standard deviation

### Reliability for other measures

Internal consistency and test–retest reliability of the SQQ-C and the PHQ-4-C were reported in Additional file 1: Table S3. Briefly, ordinal Cronbach’s alpha values ranged from 0.737 to 0.904, ordinal McDonald's omega values ranged from 0.747 to 0.904; ICCs ranged 0.632 to 0.797 for the global scale, and its subscale. Regarding structural validity, some details of which were reported elsewhere [26, 55], the SQQ-C and the PHQ-4-C respectively exhibited stable two-factor solution and favorable fit indices.

## Discussion

The aim of this study was to translate and adapt the RU_SATED scale for use in Chinese and validate the RU_SATED-C scale to provide preliminary reliability and validity when used for assessing sleep health in a Chinese population. The methodology used was similar to that used in the various languages’ validation studies of the RU_SATED scale, in Portuguese (2018), English (2019), Spanish (2020), French (2021), and Japanese (2022) [9, 16–19]. Admittedly, this is the first study to assess the psychometric performance of the RU_SATED in a Chinese population. This instrument demonstrated adequate measurement properties when used with Chinese healthcare students.

Specifically, model fit indices produced by CFAs indicated that a single-factor structure fit the data well at two time points, similar to that of Portuguese, Spanish, and French studies [16–18]. Note, however, that some significant distinctions in translations exist. For example, the Portuguese version utilized a five-point Likert scale, and the Spanish version was adapted from the original five-item SATED scale. Our observed single-factor model differed from the two-factor model obtained in the English and Japanese studies [9, 19]. It is important to note that there are differences between the two-factor models obtained in English [Factor 1 (Sleep Quality & Quantity): Satisfaction, Efficiency, Duration; Factor 2 (Circadian Rhythm): Regularity, Alertness, Timing] and Japanese [Factor 1 (Quality & Quantity): Satisfaction, Alertness, Duration; Factor 2 (Circadian): Regularity, Efficiency, Timing]. In the French validation, the CFA showed an acceptable fit for both the one-factor and two-factor structures (in common with the English version); however, the fit was slightly better for the latter [18]. Our data were well approximated by a single-factor model across both testing occasions and also supported strict factorial invariance across time. The data supported the two-factor models found in the Japanese and English samples and resulted in a better fit for the two-factor model previously found in the Japanese version and a slightly worse fit for the two-factor model found in the English version, respectively. One underlying reason is that both China and Japan belong to Oriental cultures, and they may have similar understandings of sleep health. However, there may be significant differences in the understanding of sleep habits between Oriental and Occidental cultures, such as their siesta habit and sleeping partners. Asians were found to perceive sleep problems more often than individuals of the Americas [56], perceived a weaker relation between sleep and physical health, and had a significantly shorter ideal amount of sleep [57]. More research is needed not only to replicate these studies, but to learn more about sleep health constructs.

Convergent and divergent validity were assessed with the SQQ-C and the PHQ-4-C, revealing satisfactory correlations, all in the expected directions. To establish convergent validity, ideally a multidimensional sleep scale would be used; however, since there is no scale that meets this requirement, a recently developed sleep quality questionnaire that is considered to partially overlap in terms of underlying constructs was adopted as a reference. The SQQ-C and the RU_SATED-C scale total scores were moderately correlated, while the correlation between the PHQ-4-C and the RU_SATED-C scale total scores was weak. For internal consistency, ordinal Cronbach’s alpha between the two testing times was slightly better than that of the English (0.64) and French (0.57) versions [9, 18], and were considerably lower than that of the Japanese (0.758), Spanish (0.77), and Portuguese (0.85) versions [16, 17, 19]. One potential reason behind such discrepancies is the small number of items (six) and three-point Likert-type response choices, which are known to decrease alpha. A second explanation for the lower internal consistency values might be related to the multifaceted nature of sleep health and cultural differences. The duration of sleep, sleeping locations, baby sleeping practices, ideology about napping, and more are all influenced by differences in cultures [56–59]. Cultural differences in sleep habits have a bearing on sleep health dimensions. Given some deficiencies of alpha, omega might be a practical alternative [60]. While no prior studies reported McDonald’s omega, our results confirmed that omega values tended to outperform the alpha values.

The present translation, adaptation, and validation of the RU_SATED scale for use in Chinese have notable strengths and limitations. First, our study provides initial evidence of the transcultural validation of the RU_SATED scale with support in the form of acceptable psychometric performance of the RU_SATED scale in Chinese, especially in terms of longitudinal measurement invariance and test–retest reliability. Second, the ordinal nature of item-level response choices was fully considered using McDonald's omega to evaluate internal consistency. Third, the RU_SATED scale is a brief, simple, and versatile assessment tool—its translation and adaptation to Chinese represent an important step toward universal assessment of sleep health. In addition to strengths, several limitations need to be acknowledged. First, the lack of objective measures of sleep, specifically regarding sleep timing and efficiency, which are assessed with the RU_SATED scale, may be considered potential limitations. However, objective measures of sleep can be impractical and expensive and thus infeasible for many large-scale studies. Although we did assess the convergent and divergent validity of the RU_SATED scale by comparing scores to another sleep-related scale (e.g., SQQ) and a scale not assessing sleep (e.g., PHQ-4), future studies should examine the associations with other measures of sleep health (e.g., SHI). Second, the low internal consistency (Cronbach’s alpha and McDonald’s omega) and the short-interval test–retest are two limitations of the study, perhaps restricting its ability to open practical prospects. Adding item(s) about sleep health behaviors [8] and scoring changes need to be considered as well. Third, another potential problem with generalizability from this sample is the restricted age range (minimum 17 to maximum 31 years, median = 20, interquartile range = 1). Finally, only a single cohort of healthcare students was used in this validation study. Participants, given their training, had unique medical knowledge, which may have led decrease generalizability. Importantly, traditions, cultural values, and local conditions and environments can influence sleep practices and attitudes. Therefore, future studies should further evaluate the measurement properties of the RU_SATED-C scale in additional validation studies, such as validating in community residents or a nationally representative sample.

## Conclusion

We cross-culturally adapted and validated the RU_SATED scale for use in Chinese-speaking samples. This represents an important step in continuing efforts to promote healthy sleep and confirms promising measurement properties including longitudinal measurement. The RU_SATED-C scale appears to be an easy-to-use and valid instrument for the measurement of multidimensional sleep health in healthcare students. Use of the RU_SATED-C scale may begin to raise awareness of sleep health and could pave the way for important efforts to promote healthy sleep.

## Supplementary Information

**Additional file 1: Table S1.** Means, standard deviations, skewness, kurtosis and missing of scores on items and the RU_SATED-C scale at T1 and T2 (N = 911). **Table S2.** The correlation matrix of the RU_SATED-C scores on inter-item and item-total, and with the SQQ-C and the PHQ-4-C scores on subscales and global scale (N = 911). **Table S3.** Internal consistency and test-retest reliability of the SQQ-C and the PHQ-4-C at T1 and T2.

## Data Availability

Permission to use the RU_SATED scale should be requested to Prof. Daniel J. Buysse. All rights related to the RU_SATED-C scale are reserved by the University of Pittsburgh. All data generated and/or analyzed during this study are not publicly available due to restrictions imposed by the ethics committee. The dataset supporting the conclusions is available upon reasonable request to the corresponding author.

## Abbreviations

CFA: confirmatory factor analysis
CFI: comparative fit index
DSS: Daytime Sleepiness Subscale
DWLS: mean and variance adjusted diagonally weighted least squares
GAD-2: Generalized Anxiety Disorder-2
ICC: intraclass correlation coefficient
LCFA: longitudinal confirmatory factor analysis
LMI: longitudinal measurement invariance
PHQ-4: Patient Health Questionnaire-4
PHQ-2: Patient Health Questionnaire-2
RMSEA: root means square error of approximation
RU_SATED: Regularity, Satisfaction, Alertness, Timing, Efficiency, Duration
SDS: Sleep Difficulty Subscale
SQQ: Sleep Quality Questionnaire
TLI: Tucker–Lewis index
